# Exploiting *RAG1*-deficient chickens to construct a single-cell transcriptional atlas of chicken heterophils during inflammation

**DOI:** 10.3389/fimmu.2026.1930258

**Published:** 2026-10-02

**Authors:** Seung Je Woo, Jaeryeong Kim, Minseok Seo, Jae Yong Han

**Affiliations:** 1 Department of Agricultural Biotechnology and Research Institute of Agriculture and Life Sciences, Seoul National University, Seoul, Republic of Korea; 2 Department of Computer Science and Software Engineering, Korea University, Sejong, Republic of Korea

**Keywords:** chicken, emergency myelopoiesis, heterophil, inflammation, maturation, single cell RNA sequencing

## Abstract

**Introduction:**

Avian heterophils naturally lack myeloperoxidase (MPO), making chicken a unique evolutionary model for investigating MPO-independent innate immune strategies. However, the molecular programs governing heterophil maturation and activation remain largely unexplored.

**Methods:**

Using single-cell RNA sequencing in a *RAG1*-deficient chicken model, we mapped heterophil maturation during emergency granulopoiesis and identified eight transcriptionally distinct subsets (H0–H7). We inferred a developmental trajectory spanning transcriptionally distinct heterophil subsets, from early subsets enriched for genes associated with translational activity, oxidative phosphorylation, and survival-related programs to terminal subsets enriched for effector-associated gene signatures.

**Results and Discussion:**

Unlike mammalian neutrophils, heterophils did not sequentially deploy canonical granule gene expression; instead, maturation was marked by progressive *MMP9* upregulation. Transcriptional profiling revealed a prominent enrichment of nitric oxide signaling pathway, with basal level of NADPH oxidase expression. Trajectory analysis indicated that cells transitioned from interferon-stimulated gene (ISG)-dominant states to effector profiles capable of degranulation, phagocytosis, and extracellular trap formation. Terminal subsets showed exhibited transcriptional signatures associated with enhanced extracellular matrix remodeling, acute phase responses, and nutritional immunity. Together, these findings provide a detailed single-cell atlas of avian heterophils, offering valuable evolutionary insights into the developmental trajectories and transcriptional heterogeneity of innate immune cells in a naturally MPO-independent species.

## 1 Introduction

Heterophils act as first-line responders that exhibit rapid chemotaxis, efficient phagocytosis, and robust pro-inflammatory cytokine production as the principal granulocytic effectors of the avian innate immune system and functional analogs to mammalian neutrophils (1, 2). Avian heterophils naturally lack myeloperoxidase (MPO), providing a unique and highly relevant biological model to dissect MPO-independent antimicrobial mechanisms against foreign pathogens (3, 4). During acute infection or severe inflammation, a process analogous to mammalian emergency granulopoiesis rapidly increases heterophil output, releasing both mature and immature cells into the blood and inflamed tissues (5). Although the specific transcriptional program driving this emergency response remains incompletely defined in chickens compared to the well-characterized C/EBPβ-centered mammalian program (6), strong inflammatory stimuli clearly drive massive heterophil recruitment and granuloma formation (4, 7). Consequently, profiling emergency granulopoiesis in this avian heterophils offers an unprecedented opportunity to map the fundamental molecular strategies governing MPO-independent innate immunity. However, gene programs of activated chicken heterophils at molecular level remain poorly known.

It stems from the challenges that heterophils are scarcely captured at the single-cell level in previous studies, limiting in-depth transcriptomic analysis of their heterogeneous functional states (8, 9). In murine model, severe inflammatory stress triggers emergency granulopoiesis, a process characterized by the rapid peripheral expansion of transcriptionally distinct, immature neutrophils to combat immune threats (10–12). We previously demonstrated that *RAG1*-deficient chicken spleens exhibit hyperinflammation mediated by reprogrammed myeloid-lineage cells and natural killer (NK) cells through single cell RNA sequencing (scRNA-seq) analysis (13). Because *RAG1* deficiency creates a state of systemic inflammation due to the absence of adaptive lymphocytes, we hypothesized that this environment drives a compensatory, emergency granulopoiesis-like responses in chicken heterophils, distinct from acute infection models. Investigating these reprogrammed myeloid populations will uncover gene signatures that drive heterophil-mediated inflammation and define their maturation pathway by tracking immature heterophils released in response to inflammatory cues.

In this study, we aimed to delineate the specific non-oxidative inflammation programs that drive pathological tissue responses in avian heterophils by comparing wild-type and *RAG1*-deficient heterophils across their maturation trajectory. We identified eight heterophil subsets with distinct transcriptional signatures. Seven subsets were markedly expanded in *RAG1*-deficient spleen while one predominated in wild-type spleen, reflecting transcriptomic features associated with emergency myelopoiesis under a hyperinflammatory environment. Maturation gene modules revealed sequential transcriptional shifts in proliferation, metabolism, and migratory capacity. Unlike mammalian neutrophils, granule genes were not sequentially induced; instead, *MMP9* was gradually upregulated, identifying it as a unique developmental marker of chicken heterophils. Strikingly, heterophils maintained basal NADPH oxidase expression while upregulating *NOS2*, indicating a transcriptional activation on nitric oxide (NO) pathway rather than oxidative burst-associated program. By comparing wild-type and *RAG1*-deficient heterophils across their maturation trajectory, we delineate the unique inflammatory transcriptional programs of avian heterophils.

## 2 Materials and methods

### 2.1 Generation of *RAG1*^-/-^ chickens

The *RAG1* knockout chicken was established in a previous study (14). Briefly, cultured White Leghorn (WL) primordial germ cells (PGCs), originally retrieved from the male gonads at embryonic day 6 by magnetic-activated cell sorting (MACS) were maintained in knockout DMEM (Thermo Fisher Scientific, Waltham, MA, USA) supplemented with 20% FBS (Hyclone, Logan, UT, USA), 2% chicken serum (Millipore Sigma, Burlington, MA, USA), 1x nucleosides (Millipore Sigma), 1x nonessential amino acids, 1x ABAM, β-mercaptoethanol, 10mM sodium pyruvate, 2mM L-glutamine, and human basic fibroblast growth factor (10 ng/ml; Millipore Sigma) at 37 °C with an atmosphere of 5% CO_2_ and relative humidity of 60-70%. The PGCs were transfected with 2 μg of CRISPR plasmids and 2 μg of donor plasmids suspended in 1 ml of Opti-MEM (Thermo Fisher Scientific) using the lipofectamine 2000 reagent (Thermo Fisher Scientific); this transfection mixture was added to 1 x 10^5^ PGCs. After chemically selection of transfected PGCs by exposure to G418 (300μg/ml) for up to 7 days, more than 3,000 genome-edited WL PGCs were microinjected into the dorsal aorta of Korean Ogye (KO) recipient embryos from Hamburger Hamilton (HH) stage 14–17 through a small window on the incubated eggs. After sealing the egg window with paraffin film, the eggs containing recipient embryos were incubated until hatching. The hatched chicks were raised until sexual maturation, and recipient KO roosters whose sperm was positive on WL-specific PCRs were used for testcrossing with wild-type WL hens. Genomic DNA was isolated from the feather pulp of individual progeny, and the genomic region containing the endogenous *RAG1* locus that was disrupted with the donor sequence was analyzed using specific primer sets (**Supplementary Table 1**) (14). The 1-week-old progenies of previously established *RAG1* disrupted chickens were used in this study. Management and experimental use of chickens were approved by the Institute of Laboratory Animal Resources, Seoul National University, South Korea (SNU-250226-4-1). The chickens were housed in a conventional environment under controlled, hygienic conditions and were provided with sterilized water and feed.

### 2.2 Flow cytometry analysis

A single cell suspension was prepared from spleens as previously described (14). After washing with PBS, 1 x 10^6^ cells were stained for 40 mins on ice using specific antibodies targeting for identifying leukocytes (anti-CD45), B cells (anti-Bu-1), macrophage/monocytes (KUL01) along with isotype control antibodies as the fluorescence minus one (FMO) control (**Supplementary Table 2**). The heterophils were defined as CD45^+^ Bu-1^-^ KUL01^-^ cells outside of lymphocyte gating with high granule components determined by high SSC value (15). The transdifferentiated heterophil-macrophage cells were defined as CD45^+^ Bu-1^-^ KUL01^-^SSC^High^ cells. Fluorescence of 100,000 total live cells was recorded using a FACSCalibur cytometer (BD Biosciences). Data were analyzed using FlowJo software (Treestar, Ashland, OR, USA). The FMO controls used for analyzing heterophils in this study are shown in **Supplementary Figure 1**.

### 2.3 Preparation and sequencing of a single-cell library

1-week old wild-type (n=2)_and *RAG1^-/-^* chicken (n=2) spleens were collected, and a single cell suspension was prepared according to previous study (13). Single-cell libraries were prepared by Macrogen (Seoul, Korea) using the Chromium controller and the 10x Chromium Next GEM Single Cell 3’v3.1 protocol (10x genomics, genomics document CG000204 Rev D; 10x Genomics, Pleasanton, CA, USA) as previously described (13). Then, the libraries were sequenced according to the read length [R1(28) - I1(8) -R2(91)] using the Illumina HiSeq X ten platform (Illumina, San Diego, CA, USA), as described in the user guide. The number of sequenced cells per library was 7,367 for wild-type chickens and 8,853 for *RAG1^-/-^* chickens.

### 2.4 Primary single-cell sequencing analysis

Raw single-cell sequencing reads were processed with Cell Ranger v6.1.2 (10× Genomics). Raw BCL files generated on the Illumina HiSeq platform were first demultiplexed into FASTQ format using “cellranger mkfastq.” The resulting FASTQ files were subsequently processed with “cellranger count” against the chicken reference genome (GRCg7b). The stability of the reference genome and gene annotation was evaluated using the NGS applicable index (16). Subsequent downstream analyses based on the generated raw count matrices (barcode.tsv, feature.tsv, and matrix.mtx) were primarily conducted using the Seurat R package (v5.4) (17). Matrices generated from the *RAG1*-deficient and wild-type chicken samples were combined into a single dataset using Seurat’s merge function. The proportion of mitochondrial gene expression was then calculated based on a total of 13 mitochondrial genes, including *ND1*, *COX1*, and *CYTB* (**Supplementary Table 3**). Using these metrics, cells were filtered according to the criteria nFeature_RNA > 200 & percent.mt < 10 & nCount_RNA > 500 (**Supplementary Figure 2**). After applying log normalization, PCA was performed on 2,000 variable genes identified using variance stabilizing transformation. Based on these results, an optimization analysis was conducted using the Uniform Manifold Approximation and Projection (UMAP) algorithm to determine the optimal clustering configuration. Specifically, the parameters were optimized to maximize the overall silhouette score across all clusters by varying the resolution parameter from 0.1 to 0.9 and the number of principal components used from 10 to 50 (**Supplementary Figure 3**). Following clustering, statistical hypothesis testing was performed to identify genes that were specifically upregulated in each cluster compared to other remaining cells using the Wilcoxon rank-sum test with the parameters min.pct = 0.25 and logfc.threshold = 0.25, and the final cell clusters were annotated based on these results.

### 2.5 Analysis to characterize heterogeneity in heterophils

Among the cells initially clustered and annotated, those identified as heterophils were subset for re-clustering. The clustering procedure was performed in the same manner as in the primary clustering analysis, with the exception that the resolution parameter (0.2) and the number of principal components used (20 PCs) differed based on silhouette score-based optimization. Statistical hypothesis testing to identify genes specifically upregulated in each heterophil sub-cluster was likewise performed using the Wilcoxon rank-sum test. Trajectory inference for heterophils was conducted using Slingshot (v2.18) (18). In this analysis, H5 was designated as the starting cluster of the trajectory based on the expression of cell maturation markers as its gene expression pattern showed the most immature features. Statistical hypothesis testing to identify genes associated with the pseudotime inferred by Slingshot was subsequently performed using TSCAN (v1.48) (19). In this study, a False Discovery Rate (FDR)-adjusted p-value of ≤ 0.05 after adjustment using the Benjamini & Hochberg method (20) was used as the cut-off for significance.

### 2.6 GO terms and KEGG pathway enrichment analysis

Significantly upregulated genes (adjusted p<0.05) were subjected to Gene Ontology (GO) terms, and Kyoto Encyclopedia of Genes and Genomes (KEGG) pathway enrichment analyses using DAVID (v202314) (21, 22). Significantly enriched GO terms and KEGG pathways were selected using an FDR-adjusted *P-*value cutoff of 0.05.

### 2.7 Quantitative real time PCR

Total RNA was extracted from the wild-type or *RAG1^-/-^* chicken spleens using Trizol Reagent (Thermo Fisher Scientific) and then reverse-transcribed using the Superscript III First-strand Synthesis System (Thermo Fisher Scientific). The cDNAs were subjected to qRT PCR using primer sets designed on the Primer3Plus website (https://www.primer3plus.com/index.html) (**Supplementary Table 1**). All reactions were performed under the conditions described in a previous study (13). Each test sample was run in triplicate for biological replications. Relative quantification of target gene expression was normalized to the *CSF3R*, as a proxy for heterophil abundance during stress condition (23). This approach is consistent with previous studies that utilize lineage-specific reference genes, such as *BAFFR*, for normalizing expression data in B cells (14).

### 2.8 Statistical analysis

Statistical analysis was performed using GraphPad Prism software (GraphPad Software, USA). Significant differences between groups were determined using a paired t-test and two-way ANOVA with Bonferroni’s multiple comparison test. A value of *P* < 0.05 was statistically significant.

## 3 Results

### 3.1 High-resolution atlas of splenic chicken leukocytes

We previously showed that myeloid-lineage cells were largely recruited in the *RAG1*-deficient chicken spleen and contributed to severe inflammation, reflecting the extramedullary myelopoiesis (13). To investigate whether chicken heterophils were significantly recruited in the *RAG1*-deficient chicken spleen, we compared the portion and number of spleen heterophils between 1-week-old wild-type and *RAG1*-deificent chicken. The Bu-1^-^KUL01^-^SSC^High^ heterophils were significantly increased in *RAG1*-deficient chicken (**Figures 1A, B**).

**Figure 1:**
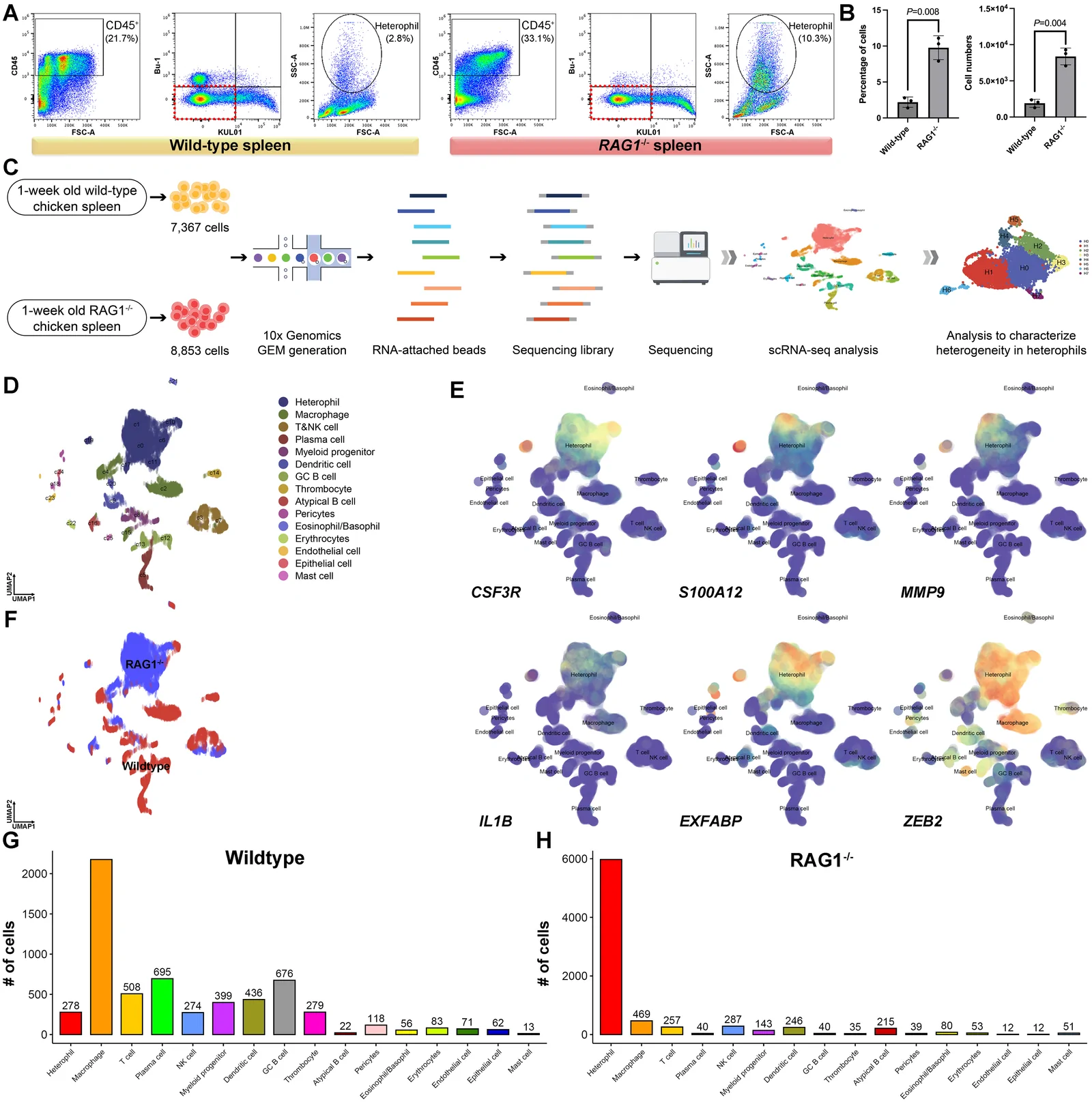
Annotation of 1-week-old chicken spleen leukocytes. **(A)** Flow cytometric analysis of heterophil populations in 1-week-old wild-type and *RAG1*-deficient spleens. Heterophils were identified as highly granular CD45^+^Bu-1^-^KUL01^-^ cells. **(B)** Quantification of heterophil percentages and absolute numbers, showing significant upregulation in *RAG1*-deficient spleens. **(C)** Overview of the scRNA-seq experimental workflow. **(D)** UMAP plots of wild-type and *RAG1*-deficient splenocytes with annotated cell clusters. **(E)** Expression of key marker genes used to identify heterophils. **(F)** Comparison of leukocyte proportions between wild-type and *RAG1*-deficient spleens, highlighting the massive expansion of heterophils in the *RAG1*-deficient model. Composition of leukocytes in **(G)** wild-type and **(H)** *RAG1*-deficient chicken spleen.

For in-depth analysis of recruited heterophils in *RAG1*-deficient chicken spleen, scRNA-seq was performed as previously reported (13). The 7,367 wild-type and 8,853 *RAG1*-deficient viable cells were sequenced using a 10x Chromium system (**Figure 1C**). Merged data were dimensionality reduced using UMAP, followed by cell population annotation based on their gene marker expression (**Figure 1D**; **Supplementary Table 4**). The gene marker expression for each leukocyte annotation is presented in **Supplementary Figures 4–18**. Cluster 5 (c5) was identified as plasma cells based on expression of *JCHAIN*, *PRDM1*, *XBP1*, and *TXNDC5* (**Supplementary Figure 4**). c12 and c13 were annotated as germinal center (GC) B cells, characterized by expression of *PAX5*, *CD79B*, and *Bu-1* (**Supplementary Figure 5**). c16 also expressed the B cell marker *Bu-1* and was selectively expanded in *RAG1*-deficient chickens. Thus, we designated it as atypical B cells (**Supplementary Figure 6**) (24). c3, c7 were annotated as T & NK cells based on overlapped expression of T cell markers (*CD3D*, *CD3E*, *ZAP70*, *TRAT1*, *TCF7*, *CD247*, *CD4*, *CD28*) (**Supplementary Figure 7**) and NK cell markers (*EOMES*, *IL2RB*, *CD8A*, *PRF1*, *GZMA*, *GNLY*, *IL2RA*, *ID2*, *FASLG*) (**Supplementary Figure 8**) (13). Dendritic cells corresponded to c9 and c20, marked by *XCR1*, *IRF8*, *DNASE1L3*, and *DMA* (**Supplementary Figure 11**), while macrophages mapped to c2, c4, and c15, expressing *TIMD4*, *MARCO*, *CSF1R*, *C1QA*, *C1QB*, and *C1QC* (**Supplementary Figure 13**).

Although proteomic comparison of splenic heterophils and macrophages identified proteins exclusively expressed in heterophils (25), reliable markers for distinguishing heterogenous heterophil subsets from macrophages remain unavailable (15). c0, c1, c6, c10, c11, c17, and c19 were initially designated as candidate heterophil clusters based on expression of *CSF3R*, *S100A12*, *MMP9*, *IL1B*, *EXFABP*, and *ZEB2* (**Figure 1E**). Notably, c0 and c1 also significantly upregulated *MMR1L4*, a chicken macrophage marker (**Supplementary Table 4**). However, c1 did not significantly express the canonical complement-associated macrophage markers genes (*C1QA*, *C1QB*, *C1QC*), whereas c0 expressed these genes at lower level than the other macrophage clusters. Moreover, both c0 and c1 lacked expression of other definitive chicken macrophage markers, such as *TIMD4* and *MARCO* (26) (**Supplementary Table 4**). The existence of populations with both heterophil- and macrophage-associated transcriptional features suggests that these subsets may have been included within the KUL01^+^ cell population during flow cytometry analysis. Indeed, both the absolute number and proportion of Bu-1^-^KUL01^+^SSC^High^ cells were significantly elevated in the *RAG1*-deficient chicken spleen compared to wild-type controls, supporting the *in vivo* presence of an inflammation-driven hybrid state sharing both granulocytic and macrophagic characteristics (**Supplementary Figure 19**). To more objectively annotate c0 and c1, we re-clustered the candidate heterophil clusters (c0, c1, c6, c10, c11, c17, c19) together with macrophage clusters (c2, c4, c15) using an increased *K* value. Under these conditions, c0, c1 and adjacent clusters (c6, c10, c11, c17) did not co-cluster with the macrophage clusters c2 or c15, indicating that they are not macrophage-lineage cells. Simultaneously, c19 exhibited the highest *CSF3R* expression, and did not co-cluster with other candidate heterophil clusters, implying that it represents the classical heterophil. In contrast, the remaining candidate heterophil clusters likely represent immature or atypical myeloid populations that share transcriptional features of both heterophil and macrophages but remain distinct from definitive macrophages (**Supplementary Figure 20**). Although the annotation of closely co-clustered populations c0, c1, c6, c10, c11, and c17 as heterophils should be interpreted with caution, we included these clusters in the heterophil compartment because they may represent immature or atypical cells along the heterophil lineage. This approach allowed us to capture the broader spectrum of heterophil maturation and inflammatory gene signatures in the absence of *RAG1* for downstream analyses.

In the *RAG1*-deficient chicken spleen, adaptive lymphocytes were largely absent, while the heterophil and heterophil-macrophage population was substantially expanded (**Figure 1F**). Notably, macrophages predominated in wild-type spleens (**Figure 1G**), whereas heterophils and heterophil-macrophage were markedly expanded in *RAG1*-deficient spleens (**Figure 1H**).

### 3.2 *RAG1*-deficient splenic heterophils display transcriptomic features of emergency myelopoiesis

To enhance subset exploration, heterophils and heterophil-macrophage clusters were extracted from the dataset and subjected to a new dimensionality reduction and differentially expressed gene (DEG) analysis (**Figure 2A**). We included heterophil-macrophage clusters to capture entire spectrum of heterophil phenotypic plasticity. This approach resolved eight distinct heterophil subsets (H0–H7). The vast majority of these subsets (H0–H4, H6, and H7) were enriched in the *RAG1*-deficient spleen, comprising 85-100% of their respective populations. In contrast, subset H5 was almost exclusively (97%) found in the wild-type spleen (**Figures 2B, C**). We next searched for candidate markers that could distinguish each subset as definitive surface markers for chicken heterophils have been lacking to date (15). Each subset exhibited a distinct transcriptional signature: H0 was enriched for *CCL19*, *CCL21*, and *CXCR13*; H1 for *ADAM8*, *PLXNB2*, and *SLC16A3*; H2 for *MARCO*, *IL13RAP2*, and *TSPAN12*; H3 for *GRID2*, *CRHR2*, and *GHR*; H4 for *ENPP3*, *PLP2*, and *LY86*; H5 for *PLP2*, *LY86*, and *SLC24A4*; H6 for *CXCR1*, *BMPR2*, and *ADAM28*; and H7 for *CCR7*, *LAMP3*, and *TNFRSF4*. Notably, H4 and H5 shared expression of several key markers, including *PLP2* and *LY86*, suggesting these two subsets are transcriptionally related (**Figure 2D**).

**Figure 2:**
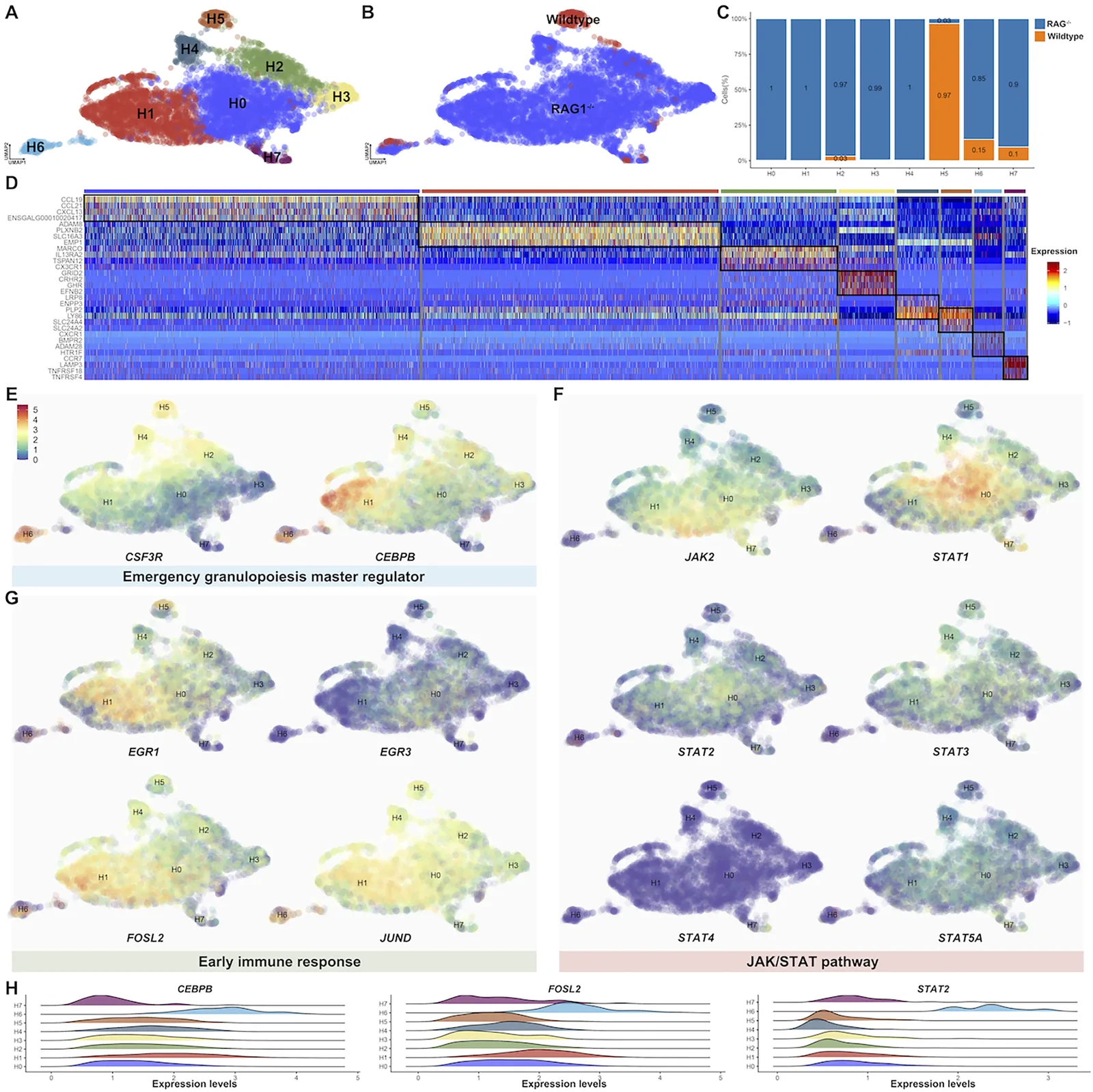
*RAG1* deficiency triggers emergency myelopoiesis-like response of spleen heterophils. **(A)** Re-clustered UMAP plot of 5,686 heterophils extracted from **Figure 1D**, revealing eight discrete subclusters (H0–H7). **(B)** UMAP distribution of wild-type (red) and *RAG1*-deficient (blue) heterophils. **(C)** Comparison of heterophil percentages between wild-type and *RAG1*-deficient chickens. **(D)** Heatmap displaying the expression of potential surface marker genes across heterophil subsets. UMAP plots showing the expression of **(E)** core transcription factors for emergency granulopoiesis, **(F)** JAK/STAT pathway components, and **(G)** early response genes. **(H)** Expression levels of core emergency granulopoiesis regulators across heterophil subsets. To minimize visualization artifacts caused by zero inflation, cells with zero expression were excluded from the visualization.

In murine model, persistent inflammation drives expansion of neutrophils in the spleen (11, 12). In this regard, marked expansion of heterophil subsets except for H5 in *RAG1*-deficient spleen implies the potential emergency myelopoiesis in the absence of *RAG1* (**Figures 2B, C**). H1 showed significant induction of the emergency granulopoiesis transcription factor *CEBPB* (6), whereas H2, H4, H5, and H6 significantly upregulated *CSF3R*, encoding the G-CSF receptor that orchestrates granulocytic demand responses (**Figure 2E**; **Supplementary Table 5**) (27). Inflammatory cytokines such as G-CSF and interferons (IFNs) drive this program and prolong neutrophil survival, in part via enhanced JAK/STAT signaling (27). Consistent with this, JAK/STAT pathway components (*JAK2*, *STAT1/2/3/5A*) were significantly upregulated across the *RAG1*-deficient dominant subsets (H0, H1, H4, H6, and H7) (**Figure 2F**; **Supplementary Table 5**). Immediate-early genes acting as first-wave responders during granulocytic commitment (28) were also robustly induced, with specific enrichment of *EGR1* and multiple AP-1 family members (*JUN*, *JUND*, *FOSL2*) in H1, *EGR3* in H0, and *FOSL2* in H6 (**Figure 2G**; **Supplementary Table 5**). Furthermore, among the cells expressing emergency granulopoiesis regulators (*CEBPB*, *FOSL2*, *STAT2*), the level of expression was highest in subset H6 (**Figure 2H**). Together, these data indicate that hyperinflammation in the absence of *RAG1* activates a robust emergency myelopoiesis-like program in chicken heterophils.

### 3.3 Single-cell transcriptomics defines immature chicken heterophil stage

In mammals, immature neutrophils are released from the bone marrow during inflammation (29), and our data indicate a comparable emergency myelopoiesis-like program in chicken heterophils (**Figures 2E–H**). Because definitive surface markers for immature chicken heterophils are not established, we instead focused on subsets with transcriptional hallmarks of immaturity—elevated translational activity (10) and high oxidative phosphorylation (OXPHOS) (30). Using this criterion, we designated H2, H4, and H5 as candidate immature heterophils, as they showed increased expression of ribosomal and translation factor genes (*RPL3*, *RPS2*, *EEF1A1*) (**Supplementary Figure 21A**) together with high OXPHOS genes (*NDUFS4*, *NDUFB2*, *ATP5B*) (**Supplementary Figure 21B**). Only H4 and H5 upregulated cholesterol biosynthesis genes (*HMGCS1*, *HMGCR*, *PMVK*) that support granule formation (**Supplementary Figure 21C**) (31, 32), indicating that H4 and H5 are potential immature heterophil subsets. Although transcriptionally similar to H5 (the dominant cluster in wild-type spleens), H4 (the dominant cluster in *RAG1*-deficient spleens) displayed a markedly activated profile. Compared to H5, H4 exhibited a significant upregulation of JAK/STAT pathway components (e.g., *STAT1*) (**Figure 3A**) and IFN-stimulated genes (ISGs) (e.g., *IFITM5*) (**Figure 3B**). Furthermore, H4 showed an elevated expression of inflammatory cytokines, such as *IL18* (**Figure 3C**). Together, these results indicate that the *RAG1*-deficient H4 cluster represents an activated state of the homeostatic H5 population.

**Figure 3:**
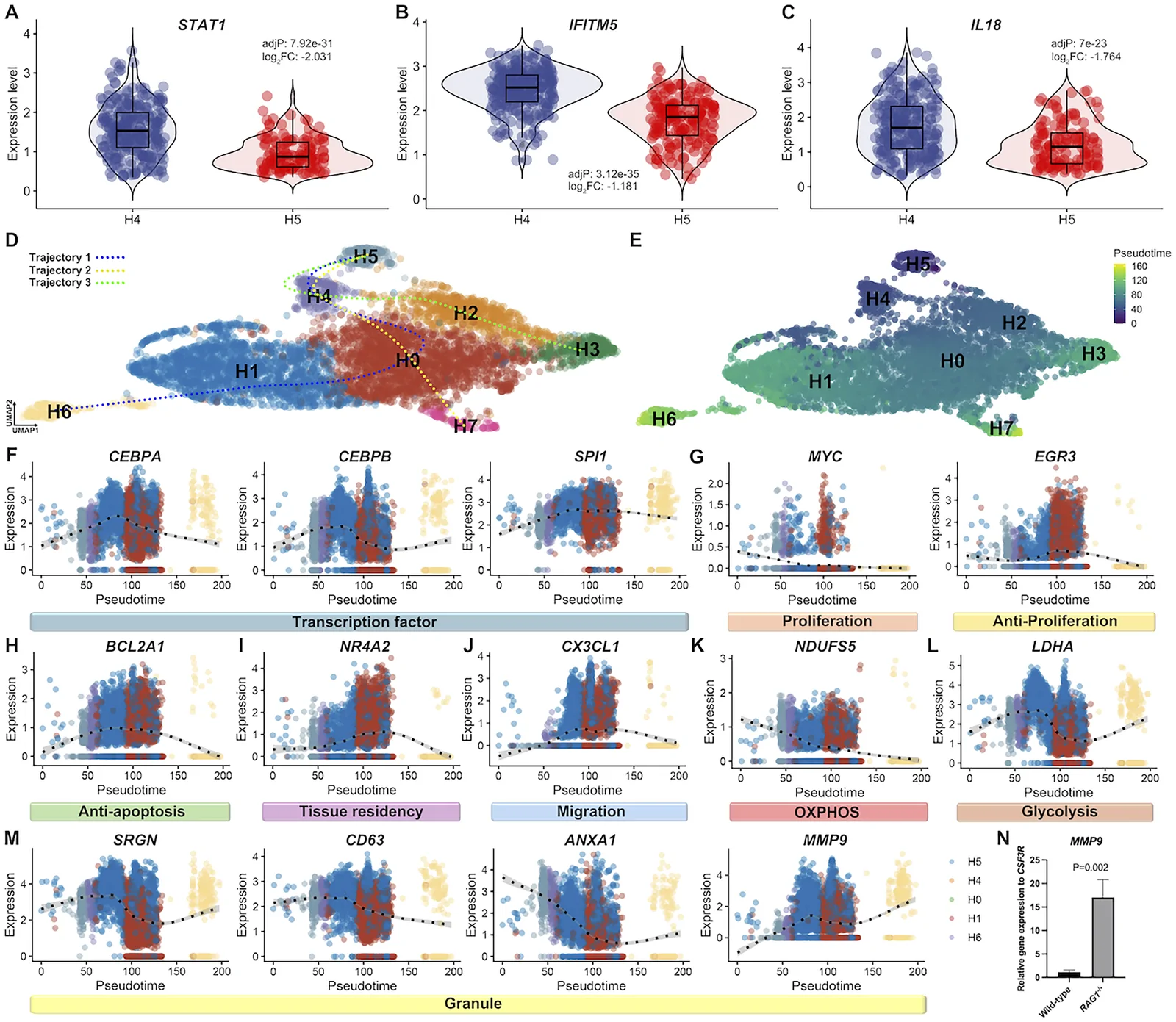
scRNA-seq of *RAG1*-deficient chicken spleens reveals the inferred maturation program of heterophils. Violin plots comparing the expression of **(A)** *STAT1*, **(B)** *IFITM5*, and **(C)** *IL18*, indicate that H4 represents an activated state of H5. **(D)** Slingshot trajectory analysis inferring the heterophil maturation pathway. **(E)** Pseudotime distribution across distinct heterophil subsets. Smoothed gene expression dynamics along pseudotime for **(F)** core transcription factors (*CEBPA*, *CEBPB*, *SPI1*), **(G)** proliferation (*MYC*) and anti-proliferation (*EGR3*), **(H)** anti-apoptosis (*BCL2A1*), **(I)** tissue residency (*NR4A2*), **(J)** migration (*CX3CL1*), **(K)** OXPHOS (*NDUFS4*), and **(L)** glycolysis (*LDHA*). **(M)** Expression profiles of primary (*SRGN*, *CD63*) and tertiary (*ANXA1*, *MMP9*) granule genes across the maturation trajectory. **(N)** The qRT-PCR showing the expression of *MMP9* normalized to *CSF3R* between wild-type and *RAG1*-deficient chicken spleen. Data are presented as the mean ± standard deviation (n = 3) and were analyzed by Student’s t-test.

### 3.4 *RAG1*-deficient spleen reveals the heterophil maturation program

We inferred heterophil maturation using Slingshot, initiating trajectories from the most immature H5 subset. This analysis predicted three potential trajectories based on transcriptional similarity: Trajectory 1: H5–H4–H0–H1–H6, Trajectory 2: H5–H4–H0–H7, Trajectory 3: H5–H4–H2–H3 (**Figures 3D, E**). The subsets positioned at the ends of these inferred trajectories (H2, H3 and H7) displayed transcriptional profiles resembling transdifferentiation-like states. These included M2 macrophage programs in H2 (**Supplementary Figure 22A**), angiogenic programs in H3 (**Supplementary Figure 22B**) and T cell–related gene expression in H7 (**Supplementary Figure 22C**), which may imply roles in tissue repair or compensation for adaptive cell loss, though independent functional validation is required. In contrast, H0, H1, and H6 were enriched for JAK/STAT signaling, inflammatory mediators, and immediate-early genes, which are transcriptional hallmarks associated with classical activation and emergency granulopoiesis-like response (**Figures 2E-H**). On this basis, we focused on the trajectory 1 (H5–H4–H0–H1–H6) as a predicted principal maturation pathway for chicken heterophils (**Figure 3D**).

We next examined the expression patterns of subset-specific transcription factors along the inferred maturation axis. The immature H4 and H5 subsets uniquely expressed granule synthesis regulator *NFE2*, and significantly upregulated early progenitor markers (*TAL1*, *PBX1*) (**Supplementary Figure 23**; **Supplementary Table 5**). In mammalian neutrophils, *CEBPA* governs early maturation and is downregulated during terminal differentiation, whereas *CEBPB* gradually increases and remains robustly expressed (33). Chicken heterophils showed a transcriptional pattern that largely mirrored this canonical C/EBP switch; *CEBPA* peaked in the early subset and declined, while *CEBPB* displayed overall upregulation although a transient decrease occurred during the intermediate stage (**Figure 3F**). *SPI1* was highly expressed in the early subset, peaked at the intermediate stage, and maintained robust expression in the later subset, a pattern consistent with established neutrophil maturation (**Figure 3F**) (34).

Along the inferred trajectory, heterophils exhibited gene expression profiles suggesting a progressive exit from the cell cycle. The proliferation marker *MYC* decreased continuously across the predicted maturation states, while the anti-proliferative gene *EGR3* increased before a decline at the final stage (**Figure 3G**), implying transcriptional features associated with cessation of proliferation. Furthermore, anti-apoptotic genes such as *BCL2A1* were transiently induced in the intermediate subsets before being downregulated in the predicted terminal stages (**Figure 3H**). Because mature neutrophils typically exhibit a short lifespan (35), we speculate that this transient anti-apoptotic signature might correlate with an intermediate tissue-resident program. Supporting this, *NR4A2*-a regulator of tissue residency and neutrophil survival (36, 37)-was also transiently induced (**Figure 3I**). Specifically, the intermediate H0 subset exhibited robust expression of anti-apoptotic genes (*BCL2A1*, *CFLAR*, *BIRC2*) and established tissue-residency markers (*CADM1*, *NR4A2*, *NR4A3*) (**Supplementary Table 5**). In contrast, the chemotactic factor *CX3CL1* increased along the trajectory, with a slight decrease in H6 (**Figure 3J**). The late-stage H1 and H6 subsets significantly upregulated genes associated with active cytoskeletal migration (*ARPC3*, *ARPC5*, *FMNL1*) (**Supplementary Table 5**). Together, these transcriptional dynamics suggest that differentiating splenic heterophils may undergo a transcriptomic shift of proliferative arrest and transit through an intermediate tissue-resident survival phase before acquiring the transcriptional hallmarks of highly migratory effector cells.

Mammalian immature neutrophils initially rely on OXPHOS before progressively shifting to glycolysis as they mature (38). To evaluate whether chicken heterophils exhibit a similar metabolic shift at the transcriptional level, we assessed their metabolic gene expression along the pseudotime axis. Consistent with the pattern seen in neutrophils, the OXPHOS-related gene *NDUFS5* was progressively downregulated, while the glycolysis-related gene *LDHA* was upregulated along the trajectory (**Figures 3K, L**). Furthermore, both the late-stage H1 and H6 subsets exhibited significant upregulation of key glycolytic genes, including *HK1*, *PFKFB4*, and *LDHA* (**Supplementary Table 5**). These results indicate that chicken heterophils display a conserved transcriptional shift from OXPHOS to glycolysis-associated programs during their predicted terminal maturation.

### 3.5 Classical neutrophil granule classification does not apply to the inferred trajectory of chicken heterophils

In mammalian neutrophils, granule biosynthesis is strictly tied to specific developmental stages (“targeting by timing”), proceeding sequentially through primary, specific, tertiary, and secretory granules (39, 40). However, most canonical mammalian granule genes—including those defining primary (*MPO*, *ELANE*, *CTSG*, *PRTN3*, *AZU1*), specific (*LNX2*, *MMP8*, *CAMP*), and secretory (*FCGR3*, *MMP25*, *VAMP2*, *STXBP4*) granules—were not differentially expressed across chicken heterophil subsets (**Supplementary Table 5**). Only a few primary and tertiary granule genes showed differential regulation. The primary granule marker *CD63* was progressively downregulated along the inferred trajectory, consistent with mammalian models; however, *SRGN* uniquely reactivated in the predicted terminal subset (upregulated in H6), diverging from the strict progressive downregulation seen in mammals (41) (**Figure 3M**). Similarly, the expression of tertiary granule genes was discordant: while *ANXA1* was progressively downregulated, *MMP9* expression increased steadily along the inferred maturation trajectory (**Figure 3M**), contradicting the coordinated late-stage upregulation of tertiary granules typical of mammalian neutrophils (41). qRT-PCR confirmed significantly elevated *MMP9* expression in *RAG1*-deficient heterophils, consistent with their enrichment of mature heterophils compared to wild-type control (**Figure 3N**). Together, these data indicate that the classical mammalian framework of sequential granule synthesis does not fully define the transcriptional landscape of chicken heterophils, though stepwise *MMP9* induction remains a robust marker of their predicted terminal development.

### 3.6 Core inflammation gene signatures of activated chicken heterophils highlight their evolutionary reliance on non-oxidative transcriptomic program

Chicken heterophils naturally lack MPO, making them an ideal model to investigate MPO-independent, non-oxidative inflammation programs (4). The severe, chronic inflammation observed in *RAG1*^-/-^ chicken spleen is driven by the loss of mature T and B lymphocytes, which fundamentally alters the inflammatory environment and induces a robust, compensatory innate immune activation (13, 14). By comparing wild-type (resting) and *RAG1*^-/-^ (inflammatory) heterophils, we sought to investigate the transcriptional landscape of this hyperactivated state. This context allows us to observe how avian heterophils transcriptionally induce systemic inflammation naturally independent of MPO activity.

In this inflammatory context, we observed strong induction of transcripts encoding viral RNA sensors (*IFIH1*, *DHX58*, *ZC3HAV1*), IFN-responsive genes (*DDX60*, *IFIT5*), IFN receptors (*IFNAR1*, *IFNAR2*, *IFNGR2*), and IFN-inducible genes (*IFI27L2*, *IFI6*, *LY6E*). *RAG1*-deficient heterophils also upregulated JAK–STAT components (*JAK2*, *STAT1*, *STAT2*) and IRFs (*IRF1*, *IRF7*), leading to robust ISG expression (*MX1*, *OASL*, *RSAD2*) (**Figure 4A**; **Supplementary Table 6**). In parallel, activated heterophils showed heightened expression of inflammatory cytokines (*IL6*, *IL18*, *IL1RL1*) (**Figure 4B**), lipid mediator pathways (*ALOX5*, *PTGS2*, *HPGD*) regulating inflammation (**Figure 4C**), and chemokines/chemokine receptors involved in leukocyte recruitment (*CX3CL1*, *CCR2*, *CCL19*) (**Figure 4D**). Together, these data indicate that, in the context of *RAG1* deficiency, chicken heterophils exhibit a transcriptional program characterized by IFN-associated signaling, inflammatory gene expression and lipid mediator-mediated pathways.

**Figure 4:**
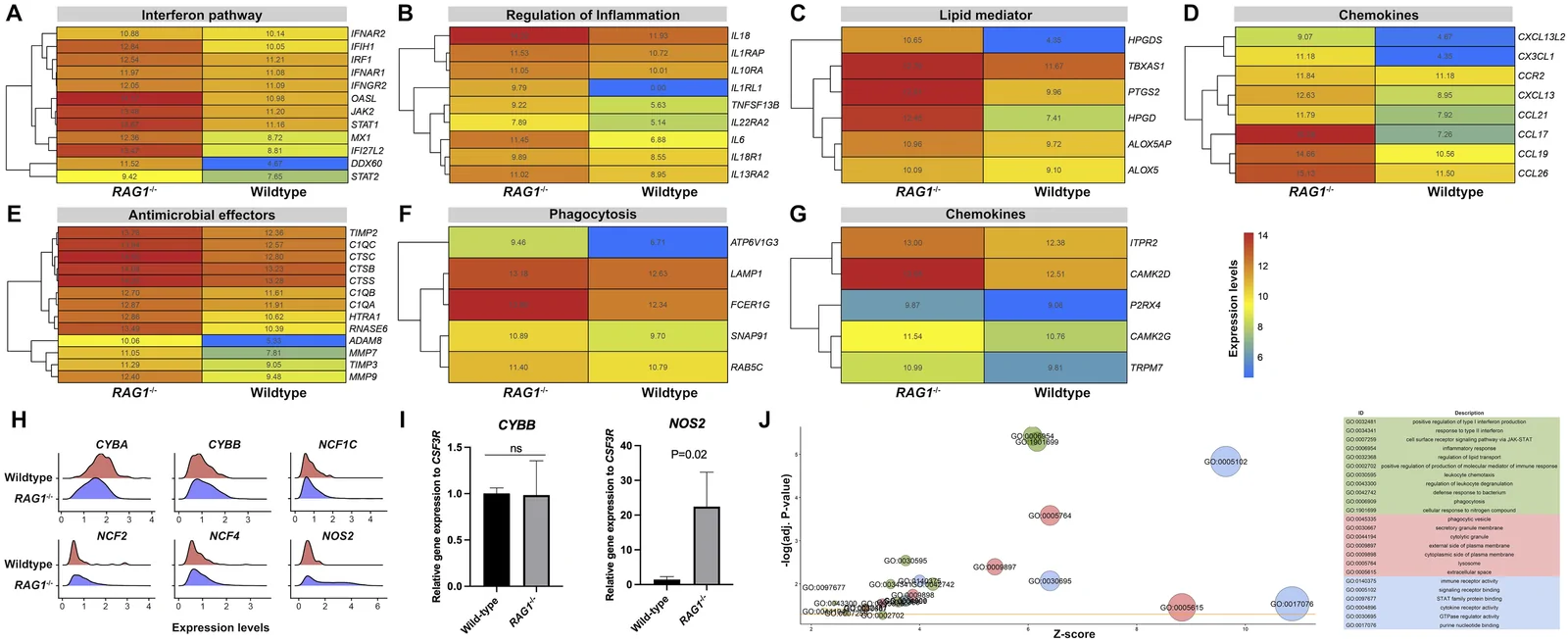
Core inflammatory gene expression of chicken heterophils. Heatmaps comparing inflammatory gene expression between resting (wild-type) and activated (*RAG1*-deficient) heterophils, categorized by **(A)** the interferon pathway, **(B)** inflammation, **(C)** lipid mediators, **(D)** chemokines, **(E)** antimicrobial effectors, **(F)** phagocytosis, and **(G)** HET formation. **(H)** Expression levels of NADPH oxidase components and *NOS2* across heterophil subsets. **(I)** The expression pattern of *CYBB* (NADPH oxidase core subunit), and *NOS2* between wild-type and *RAG1*-deficient chicken heterophils measured by qRT-PCR normalized to *CSF3R*. Data are presented as the mean ± standard deviation (n = 3) and were analyzed by Student’s t-test. ns; non-significance. **(J)** Enriched GO terms in activated heterophils.

Activated chicken heterophils upregulated transcripts encoding diverse antimicrobial effectors, including *RNASE6*, cathepsins (*CTSS*, *CTSB*, *CTSC*), matrix metalloproteinases (MMPs) (*MMP7*, *MMP9*), serine proteases (*HTRA1*, *PRSS*), and complement components (*C1QA*, *C1QB*, *C1QC*). They also increased the expression of extracellular matrix (ECM)-remodeling inhibitors, such as *TIMP2* and *TIMP3* (**Figure 4E**; **Supplementary Table 6**). In parallel, they strongly induced gene signatures associated with phagocytic machinery, encompassing scavenger receptors (*FCER1G*), Rab GTPases (*RAB5C*, *RAB7*), endocytic components (*PLCALM*, *SNAP91*), acidification factors (*TCIRG1*, *ATP6V1G3*), and lysosomal markers (*LAMP1*) (**Figure 4F**; **Supplementary Table 6**).

In neutrophils, MPO-derived hypochlorite helps initiate neutrophil extracellular trap (NET) formation by triggering Ca^2+^ signaling and chromatin decondensation (42, 43). Activated chicken heterophils showed a similar Ca^2+^-signaling profile (*ITPR2*, *CAMK2D*, *CAMK2G*) together with cathepsin family induction, implying a potential transcriptional readiness for heterophil extracellular trap (HET) formation (**Figure 4G**). Notably, transcripts encoding NADPH oxidase components were largely unchanged except for *NCF2*, indicating no broad transcriptional induction of the superoxide-generating NADPH oxidase pathway. In contrast, they significantly upregulated *NOS2*, suggesting an enrichment of NO-associated transcriptional pathways relative to NADPH oxidase-related gene programs (**Figure 4H**; **Supplementary Table 6**) (44). Indeed, qRT-PCR confirmed that while *CYBB* (core component of NAPDH oxidase) expression was not significantly altered, *NOS2* was significantly induced in *RAG1*-deficient chicken heterophils (**Figure 4I**). Furthermore, upon activation, heterophils exhibited significantly increased expression of the danger signal receptor such as *P2RX4*. This upregulation likely enhances the transcriptional capacity to sense extracellular ATP, and is associated with Ca^2+^ signaling pathways necessary to drive HET formation (**Figure 4G**) (45).

GO analysis corroborated these transcriptional patterns, revealing an enrichment for cellular responses to nitrogen compounds (GO:1901699) over traditional oxidative burst pathways. These NO-associated signatures operated alongside transcriptional programs for classical degranulation, phagocytosis (GO:0043300, GO:0006909), and purine-mediated ATP sensing (GO:0017076), all orchestrated by robust interferon and lipid-regulated inflammatory signaling networks (GO:0032481, GO:0006954, GO:0032368, GO:0002702) (**Figure 4J**).

### 3.7 Dynamic reshaping of inflammation programs along the inferred heterophil trajectory

We next examined how inflammation programs change along the inferred heterophil maturation trajectory (H4-H0-H1-H6), focusing on H0, H1, and H6. H0 showed strong induction of ISGs (*RSAD2*, *MX1*, *OASL*) (**Figure 5A**) together with JAK–STAT and IRF family transcriptional programs (**Figure 2F**). It also expressed lymphoid chemokines (*CCL19*, *CCL21*, *CXCL13*), which may reflect a compensatory response to the loss of adaptive lymphocytes (**Figure 5B**). Notably, H0 upregulated purinergic receptors (*P2RY6*), Ca^2+^-signaling components (*ITPR2*, *TRPM7*), Ca^2+^/calmodulin-dependent kinases (*CAMK1D*, *CAMK2D*, *CAMK2G*) and the histone deacetylase *HDAC2*, which is required for murine NETosis (**Figure 5C**) (46). Enriched GO terms highlighted an upregulation of IFN-mediated pathways (GO:0032481) and lymphocyte activation (GO:0046649). Furthermore, molecular function analyses revealed that these upregulated genes are involved in ATP binding (GO:0005524) and histone modification (GO:0140993)-essential processes for HET formation (**Figure 5D**). Together, these data suggest that H0 exhibits a transcriptional profile characteristic of an ISG-high, tissue-resident subset. This transcriptional signature includes a lymphocyte-recruitment chemokines, purinergic receptors, and Ca^2+^-CAMK-HDAC2 pathway-associated genes. These are consistent with pathways implicated in danger-associated molecular pattern (DAMP) sensing and extracellular trap formation; however, their functional validation is required.

**Figure 5:**
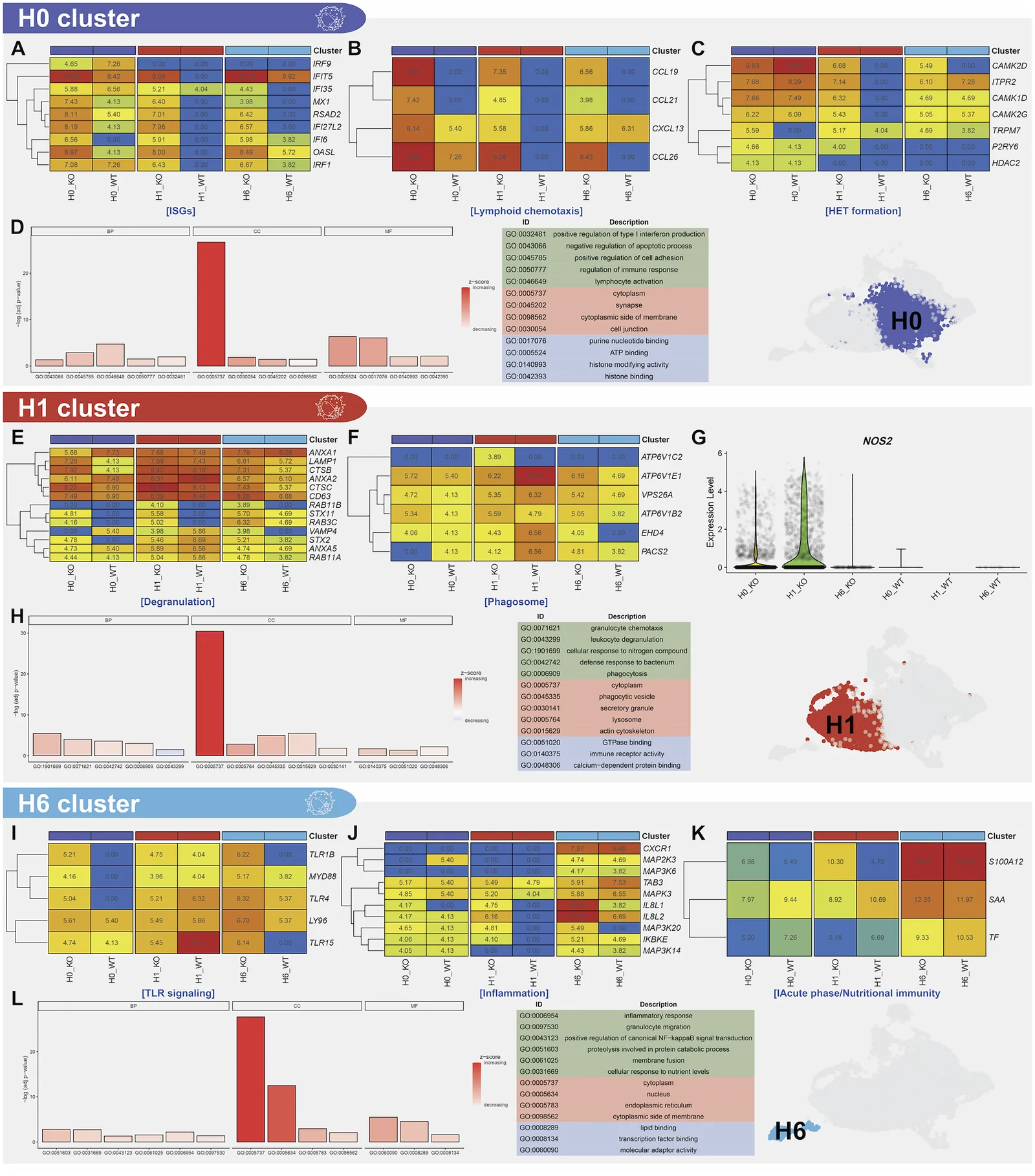
Inflammatory gene programs of chicken heterophils across maturation. Observation of differential expression patterns between wild-type and *RAG1*-deficient cells within each of the H0, H1, and H6 heterophil subclusters. Heatmaps of H0-specific gene expression for **(A)** ISGs, **(B)** lymphoid chemokines, and **(C)** HET formation. **(D)** Enriched GO terms for H0. Heatmaps of H1 functional genes related to **(E)** degranulation and **(F)** phagosomes. **(G)** Expression profile of *NOS2*, which is upregulated in H1. **(H)** Enriched GO terms for H1. Heatmaps of H6 functional signatures for **(I)** TLR signaling, **(J)** inflammation, and **(K)** acute phase responses and nutritional immunity. **(L)** Enriched GO terms for H6.

H1 was enriched for genes associated with degranulation and phagocytosis. It upregulated Rab GTPases (*RAB3C*, *RAB11A*, *RAB11B*), annexins (*ANXA1*, *ANXA2*, *ANXA5*), and SNARE components (*VAMP4*, *STX2*, *STX11*), involved in Ca^2+^-dependent granule trafficking and fusion (**Figure 5E**) (47). Transcripts for antimicrobial granule proteins (*CTSB*, *CTSC*, *CD63*, *LAMP1*) and phagosome-associated factors (*VPS26A*, *ATP6V1C2*) were also elevated (**Figures 5E, F**). Although *NCF2* and *CYBC1* were expressed, other NADPH oxidase subunits were not differentially expressed, whereas *NOS2* was strongly induced, suggesting a transcriptional reliance on NO-related pathway (**Figure 5G**; **Supplementary Table 5**). Significantly enriched GO terms highlighted leukocyte degranulation (GO:0043299), phagocytosis (GO:0006909), and responses to nitrogen compounds (GO:1901699), with the corresponding gene products primarily localized to phagocytic vesicles (GO:0045335) and granules (GO:0030141) (**Figure 5H**). Together, these features define H1 as a transcriptionally degranulating, phagocytic subset characterized by *NOS2* induction.

H6 displayed a transcriptional profile consistent with a terminally differentiated, pro-inflammatory state. It upregulated multiple TLRs (*TLR4*, *TLR1B*, *TLR15*) and the adaptor *MYD88*, together with MAPK (*MAP3K6*, *MAP3K14*, *MAP3K20*) and NF-κB pathway components (*IKBKE*, *TAB3*), and pro-inflammatory cytokines/chemokines (*IL8L1*, *IL8L2*, *CXCR1*) (**Figures 5I, J**). In parallel, H6 induced highest *MMP9* expression (**Figure 3M**), along with other ECM-remodeling enzymes (*ADAM8*, *ADAM10*) (**Supplementary Table 5**), and acute phase genes (*SAA*, *S100A12*, *TF*) (**Figure 5K**). The upregulation of *S100A12* and *TF* suggests a potential role in zinc and iron sequestration (nutritional immunity), respectively, depriving microbes of these nutrients (**Figure 5K**) (48–50). Enriched GO terms confirmed various signaling pathways for inflammatory response (GO:0006954; GO:0043123; GO:0060090), and nutritional immunity (GO:0031669) (**Figure 5L**). Thus, the transcription program of H6 appears shifted away from signatures of degranulation toward a predicted hyperinflammatory, tissue-remodeling phenotype that leverages ECM degradation, acute phase response and nutritional immunity.

**Figure 6** summarizes the inferred developmental programs and inflammatory profiles of each heterophil subset based on transcriptomic data. As heterophils mature, they exhibit gene expression consistent with halting proliferation, shifting metabolism from OXPHOS to glycolysis, and inducing migratory genes. Mid-maturation subsets upregulate anti-apoptotic genes, suggesting a tissue resident-associated transcriptomic features. Immature heterophils express programs for cholesterol synthesis and upregulate various ISGs and lymphoid chemokines. In contrast, transcriptionally mature heterophils display gene signatures of classical granulocyte antimicrobial functions, such as phagocytosis and putative HET formation, while terminally differentiated subsets specialize in gene programs for ECM remodeling, acute phase responses, and nutritional immunity, coupled with hyper-inflammation.

**Figure 6:**
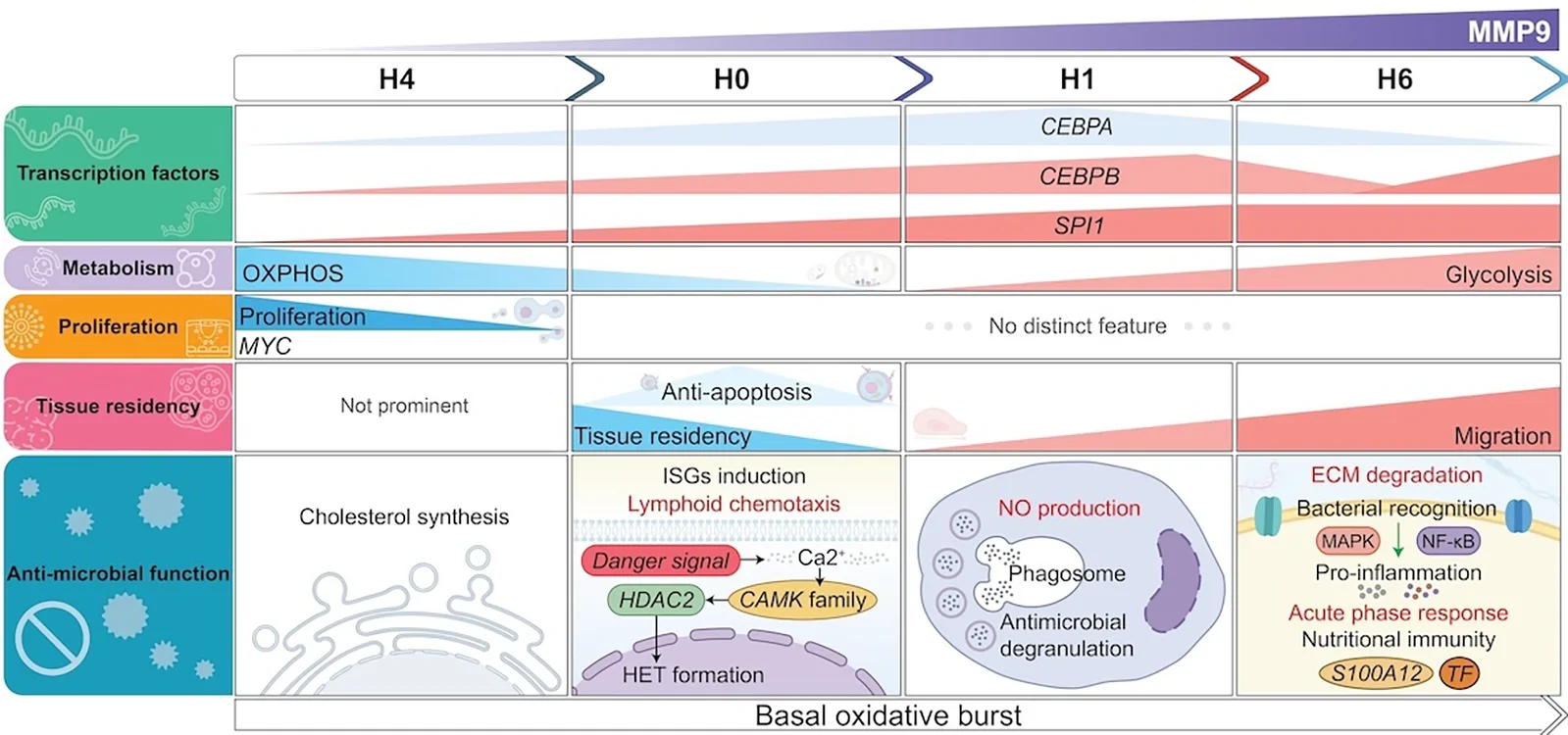
Transcriptional signatures of core developmental and inflammation-associated features across inferred heterophil maturation stages. The inferred maturation trajectory is characterized by the downregulation of proliferation-associated transcripts and a shift from OXPHOS- to glycolysis-related gene expression. Expression of the core transcription factors *CEBPB* and *SPI1* increases progressively, whereas *CEBPA* expression peaks early and declines in later inferred stages. Early-stage heterophil subsets show enriched expression of genes associated with cholesterol biosynthesis, a pathway linked to early granule formation. At the intermediate stage, the upregulation of anti-apoptotic genes coincides with tissue residency-associated transcripts in the H0 subset. This subset also exhibits enriched expression of interferon-stimulated genes (ISGs), lymphoid chemokines, purinergic receptors, and calcium signaling/*HDAC2*-associated transcripts—implicated in extracellular trap formation. In contrast, late-stage subsets (H1 and H6) upregulate transcripts associated with cell migration. The H1 subset is enriched for *NOS2* alongside gene modules related to phagocytosis and degranulation, while transcripts encoding NADPH oxidase components remain near basal levels across the trajectory. The late-stage H6 subset upregulates TLR, MAPK, and NF-κB signaling components, alongside transcripts for *MMP9*, acute-phase proteins, and nutritional immunity mediators, consistent with a robust inflammatory transcriptional state. Features highlighted in red represent transcriptional programs divergent from those of canonical mammalian neutrophils.

## 4 Discussion

The *RAG1*-deficient chicken model represents an ideal platform for comprehensive single-cell analysis of heterophils, addressing a critical technical limitation of low heterophil numbers in previous scRNA-seq studies (8, 9). However, it is essential to acknowledge that the expansion and activation of these heterophils occur within a profoundly altered immunological context. The absence of adaptive lymphocytes disrupts normal immune hemostasis, triggering compensatory extramedullary myelopoiesis-like response and chronic inflammation (13)-a phenotype similarly documented in TCR αβ knockout chickens and *RAG1*-deficient mice which exhibit significant increases in monocyte populations and granulocytes associated with inflammatory lesions (51, 52). Consequently, the heterophil heterogeneity and maturation trajectories identified in our study must be interpreted within this chronically inflamed environment. Rather than representing strictly intrinsic, steady-state biological properties, many of the identified transcriptional signatures likely represent secondary adaptions to altered cytokine networks and the compensatory innate immune requirements of the immunodeficient hosts. Thus, while our atlas captures the dynamic, hyper-activated state of avian heterophils during inflammation, future comparative studies using wild-type acute infection models will be necessary to fully disentangle developmental programs from these inflammation-induced compensatory mechanisms.

We identified heterogeneous heterophil subsets that were expanded in *RAG1*-deficient spleens, compared to wild-type spleens, a phenotype indicative of emergency myelopoiesis-like response. In response to the chronically altered immunological environment caused by the loss of adaptive lymphocytes, *RAG1*-deficient heterophils significantly upregulated JAK/STAT pathway components (27), early immune response genes (28), and the core transcription factor *CEBPB* (6), patterns that parallel mammalian neutrophil emergency granulopoiesis. Several heterophil subsets exhibited transcriptional profiles resembling transdifferentiation toward tissue-repair and compensatory phenotypes. The c0, and c1 significantly expressed the macrophage-associated marker *MMR1L4*, yet lacked other macrophage markers such as *TIMD4* and *MARCO* (26). Concurrently, they robustly maintained expression of heterophil-specific genes, including *MMP9* and *RNASE6* (25, 53). To objectively annotate c0 and c1, we re-clustered the candidate heterophil and macrophage clusters and found that c0 and c1 co-clustered with other heterophil clusters rather than with macrophage clusters. Given the lack of reliable markers for distinguishing heterogenous heterophil subsets (15), this progressive re-clustering analysis suggests that c0 and c1 predominantly exhibit transcriptomic features associated with the heterophil lineage while retaining macrophage-like characteristics. This closely mirrors the adaptive plasticity observed in mammalian neutrophils, where human and mouse neutrophils undergo partial transdifferentiation to acquire macrophage functional features under chronic inflammatory stress (54, 55). When analyzing within the heterophil subsets, we identified seven heterophil subsets (H0-H6). H2 expressed tissue-resident M2 macrophage markers, efferocytosis receptors, and anti-inflammatory regulators, aligning transcriptionally with neutrophil-to-monocyte/macrophage transdifferentiation described during inflammation (56). H3 displayed features typical of proangiogenic neutrophils implicated in neovascularization (57, 58), while H7 upregulated T cell-associated gene expression. These reprogrammed subsets likely represent transcriptional plasticity and compensatory responses of myeloid cells adapting to the absence of mature B and T lymphocytes, rather than definitive cell-fate transitions (59, 60).

Despite the presence of atypically reprogrammed profiles, we identified an inferred principal maturation trajectory progressing through H5-H4-H0-H1-H6. H4 (activated form of H5) and H5 exhibited hallmarks of immature granulocytes, including elevated OXPHOS and translational activity, consistent with mammalian immature neutrophils (38, 61–63). H4, H5 expressed progenitor-associated transcription factors (*TAL1*, *PBX1*) (64, 65) and the proliferation marker *MYC* (66). In terms of metabolic pathways, H4, H5 showed enrichment for cholesterol synthesis genes, which are required for early granule formation (31, 32). Together, these transcriptional features suggest H4, H5 represents immature heterophil populations released during inflammation, potentially analogous to mammalian promyelocytes or early myelocytes.

While core transcription factors (*CEBPA*, *CEBPB*, *SPI1*) shared expression trajectories with mammalian neutrophils (33, 34), canonical granule genes did not exhibit the classical sequential induction in chicken heterophils. Primary granule genes (e.g., *SRGN*) were reactivated in terminally differentiated H6 subset, and tertiary granule genes (e.g., *ANXA1*) were progressively downregulated—patterns opposite to those in mammalian neutrophils (41). However, *MMP9* showed gradual upregulation, serving as a robust maturation marker similar to its role in zebrafish neutrophils (67). These results suggest that the classical “targeting by timing” mammalian granule framework does not strictly govern chicken heterophil maturation at the transcriptional level, though stepwise *MMP9* induction tracks reliably with terminal development.

The H0 subset exhibited transcriptional features associated with survival, tissue-resident heterophils adapted for prolonged surveillance. H0 significantly upregulated *NR4A2* and *NR4A3*, master regulators of neutrophil lifespan (36, 68), and demonstrated robust ISG expression. This parallels mammalian ISG-high neutrophils, which suppress viral replication and amplify immune signaling upon IFN stimulation (69, 70). Notably, H0 expressed lymphoid chemokines typically produced by stromal cells, a likely compensatory adaption to orchestrate leukocyte recruitment in the *RAG1*-deficient host (71). Furthermore, H0 upregulated purinergic receptors associated with sensing extracellular ATP (72). This purinergic signature, combined with elevated calcium-dependent kinases and *HDAC2* expression, suggests H0 heterophils may be transcriptionally primed for calcium-dependent HET formation, though functional verification is necessary (73, 74).

The H1 subset exhibited transcriptional signatures of classical granulocyte maturation and inflammation. Unlike the H0 subset, H1 demonstrated a transcriptional shift from OXPHOS to glycolysis and expressed genes associated with cellular migration. Furthermore, H1 showed enriched expression for degranulation machinery including SNARE components and annexins; however, *RAB27A* was not detected. In mammalian neutrophils, Rab27a is essential for primary granule exocytosis (75), and its absence suggests heterophils may utilize distinct vesicular trafficking networks, consistent with their unique granule composition. Additionally, H1 strongly upregulated *NOS2* while NADPH oxidase components remained unchanged. Prior functional study revealed that chicken macrophages-rather than heterophils-are the dominant producers of NO (76). Thus, this *NOS2* induction in the H1 likely reflects phenotypic plasticity, where heterophils acquire macrophage-associated features in response to the hyperinflammatory *RAG1*-deficient environment (54, 55). Alternatively, this upregulation mirrors compensatory mechanisms observed in MPO-deficient mice where iNOS expression is augmented to maintain its activity (77), suggesting that maturing heterophils may leverage NO-driven pathways as a primary effector strategy during inflammation. Therefore, future functional validation is required to determine whether the H1 subset achieves macrophage-like NO output specifically during chronic inflammation, and/or whether a NO-mediated augmentation occurs in healthy birds responding to acute infection.

The H6 subset displayed a transcriptional profile consistent with a terminally differentiated, hyperinflammatory state featuring high glycolytic activity and migratory gene expression, mirroring the hallmarks of mature mammalian neutrophils (38, 78). H6 displayed robust TLR expression and downstream MAPK/NF-κB activation signatures, aligning with inflammatory amplification (79). Notably, H6 showed gene signatures of profound ECM remodeling through its maximal *MMP9* induction. While excessive MMP9 often drives pathological tissue damage in mammals (80, 81), it is also crucial for immune cell migration and tissue regeneration in zebrafish (82, 83), suggesting this remodeling may be a prominent antimicrobial strategy in non-mammalian vertebrates. Additionally, H6 expressed nutritional immunity genes, including *S100A12* (encoding MRP126) and *TF* (transferrin), to sequester zinc and iron from pathogens (49, 50). This contrasts with the reliance of mammalian neutrophils on *S100A8*/*S100A9* (calprotectin) and *LTF* (lactoferrin) expression (84–86), demonstrating that chicken heterophils achieve analogous nutritional immunity through distinct molecular effector gene expression.

High-resolution single-cell atlases of neutrophil maturation and inflammatory programs have enabled direct cross-species comparisons, revealing both conserved and divergent features. Comparative transcriptomics of zebrafish, mouse, and human neutrophils found high molecular similarity in immature stages and identified pan-species gene signatures including C/EBPβ-driven late maturation (67), and human-mouse integrated analyses demonstrated strong conservation of core transcriptional programs (87). In contrast, chicken heterophils have remained underrepresented in scRNA-seq studies; prior chicken peripheral blood leukocyte profiling captured limited numbers of heterophils and reported insufficient resolution for detailed phenotypic characterization (8). By leveraging *RAG1*-deficient chickens with expanded myeloid compartments due to compensatory innate activation, this study provides the first comprehensive transcriptomic atlas of eight heterophil subsets. We computationally reconstructed a canonical maturation trajectory (H5-H4-H0-H1-H6) and delineated the transcriptional landscape of heterophils operating in a naturally MPO-deficient immune system under chronic inflammatory stress. These findings establish a critical foundation for avian-mammalian comparative immunology, illuminating evolutionary adaptations in granulocyte biology and enabling systematic dissection of conserved versus lineage-specific innate immune features across vertebrates.

This study has several limitations. Prior studies have successfully demonstrated heterophil functions, such as ROS production (88) and HET formation (89), using bulk cellular assays. However, the primary aim of our study was to map heterogenous heterophil subsets to these specific functional modules based on single-cell transcriptional data. While confirming these distinct functional states is essential, the current lack of subset-specific antibodies prevented functional validation of the transcriptomically defined heterophil populations. Consequently, we were unable to functionally verify specific heterophil subset’s functions, such as their reliance on the NO pathway over the classical oxidative burst. Second, the proportion of atypical heterophils with macrophage-like features in *RAG1*-deficient chickens requires further *in vivo* validation. Several candidate heterophil clusters co-expressed heterophil-associated genes and the macrophage marker, *MMR1L4*, suggesting a mixed transcriptional phenotype. Although flow cytometric analysis showed an increased proportion of KUL01^+^SSC-A^High^ cells, this population may also include activated macrophages and therefore cannot definitively quantify atypical heterophils. In addition, discrepancies between cell-type frequencies measured by scRNA-seq and flow cytometry may reflect platform-specific technical limitations, particularly the limited availability of antibodies capable of resolving heterogenous heterophil subsets (90). Thus, distinguishing atypical heterophils from macrophages remains challenging in the absence of reliable heterophil-specific markers (15). Nevertheless, unbiased re-clustering analysis consistently placed the candidate atypical heterophil clusters separated from classical macrophage clusters, supporting the expansion of myeloid cells distinct from macrophages under inflammatory condition in the absence of adaptive lymphocytes. Furthermore, populations isolated based on high granularity may still contain minor fractions of eosinophils and basophils. Thus, future studies employing higher resolution sorting strategies coupled with morphological validation, such as Wright-Giemsa staining (91), will be necessary to precisely isolate and characterize these diverse heterophil subsets. Third, while we captured splenic emergency myelopoiesis-like response, characterizing the complete maturation trajectory requires investigation of bone marrow progenitors to separately investigate whether expanded heterophils are newly generated or recruited from other stie. Furthermore, the developmental continuum proposed in this study is inferred computationally through pseudotime analysis. Definitive confirmation of this maturation pathway will require *in vivo* lineage tracing or subset-specific functional assays to validate the true ontogeny and temporal progression of these transcriptionally defined heterophil states. Fourth, the absence of classical antimicrobial granule transcripts (e.g., *CATHL2*, *AvBDs*) likely reflects that these genes were already upregulated during maturation in the bone marrow. Indeed, *CATHL*, and *AvBD* genes are highly expressed in bone marrow compared to other tissues (1, 92, 93). Human neutrophils also showed granule protein mRNAs were downregulated after early maturation despite of persistence of stored proteins (94). Consequently, scRNA-seq of bone marrow is necessary to capture this early transcriptional phase in chickens. Fifth, while *CSF3R* was used as proxy for heterophil abundance in bulk spleen tissue, its expression varies dynamically across maturation states. Therefore, it does not fully control for transcriptional variability between distinct heterophil subsets. Sixth, as this study utilized spleens from 1-week-old chicken, the findings cannot be generalized across different developmental ages or tissue compartments. Seventh, the functional plasticity of heterophils should be examined under a broader range of stimuli. In the current model, *RAG1* deficiency creates a systemic inflammation (13) and compensatory reprogramming-manifesting as atypical transdifferentiation-due to the absence of adaptive immunity. While we successfully defined the classical maturation trajectory, this environment reflects a chronically activated state. Accordingly, the biological inferences drawn from this study should be interpreted cautiously and should not be overgeneralized to heterophil maturation programs or functions in other contexts. Rather, this atlas provides transcriptional signatures of both classical, terminally differentiated heterophils and distinct atypical or immature heterophil populations in the *RAG1*-deficient chicken spleen. Future scRNA-seq studies utilizing controlled infection assays in wild-type and *RAG1*-deficient embryos would refine our understanding of heterophil antimicrobial function by separating acute pathogen responses from chronic compensatory mechanisms.

## Data Availability

All data generated or analyzed during this study are included in this published article and its **Supplementary Information** files. The scRNA-seq datasets for chicken spleen during the current study are available in the GEO repository (GSE255238).
