# Supplementary material for: Exploiting RAG1-deficient chickens to construct a single-cell transcriptional atlas of chicken heterophils during inflammation
Source: Front Immunol. 2026 Oct 2;17:1930258. doi: 10.3389/fimmu.2026.1930258 (PMC13671945; doi:10.3389/fimmu.2026.1930258)
Supplement: Supplementary file 1 [file Table1.docx]

Supplementary Material

**Supplementary Figure 1**. Gating strategy to analyze heterophils in chicken spleen. Fluorescence minus one (FMO) control for CD45, Bu-1, and KUL01 were emplyoed for the investigation of heterophils.

Supplementary Figure 2. Single cell RNA sequencing quality check. The violin plot shows the distribution of nFeature_RNA, the number of unique genes detected per cell, nCount_RNA, the total number of UMI counts per cell, and percent.mt, the percentage of mitochondrial gene expression. Cells with more than 10% of mitocondrial content were filtered out to remove low-quality/dying cells.

Supplementary Figure 3. Optimization results for primary clustering. When varying the resolution parameter from 0.1 to 0.9 and the number of principal components (PCs) from 10 to 50, the highest average silhouette width of 0.43 was observed at resolution = 0.4 and PCs = 40. The silhouette width for each cluster, along with the overall average silhouette width, is visualized for this optimal parameter combination.

Supplementary Figure 4. Gene marker expression to annotate plasma cell. Analysis of gene marker expression to annotate plasma cells

Supplementary Figure 5. Gene marker expression to annotate GC B cell. Analysis of gene marker expression to annotate germinal center (GC) B cells

Supplementary Figure 6. Gene marker expression to annotate Atypical B cell. Analysis of gene marker expression to annotate Atypical B cells

Supplementary Figure 7. Gene marker expression to annotate T cells. Analysis of gene marker expression to annotate T cells

Supplementary Figure 8. Gene marker expression to annotate NK cells. Analysis of gene marker expression to annotate Natural Killer (NK) Cells

Supplementary Figure 9. Gene marker expression to annotate thrombocyte. Analysis of gene marker expression to annotate thrombocytes

Supplementary Figure 10. Gene marker expression to annotate erythrocytes. Analysis of gene marker expression to annotate erythrocytes

Supplementary Figure 11. Gene marker expression to annotate dendritic cell. Analysis of gene marker expression to annotate dendritic cells

Supplementary Figure 12. Gene marker expression to annotate myeloid progenitor. Analysis of gene marker expression to annotate myeloid progenitors

Supplementary Figure 13. Gene marker expression to annotate macrophage. Analysis of gene marker expression to annotate macrophages

Supplementary Figure 14. Gene marker expression to annotate eosinophil/basophil. Analysis of gene marker expression to annotate Eosinophil/Basophil

Supplementary Figure 15. Gene marker expression to annotate mast cells. Analysis of gene marker expression to annotate mast cells

Supplementary Figure 16. Gene marker expression to annotate epithelial cells. Analysis of gene marker expression to annotate epithelial cells

Supplementary Figure 17. Gene marker expression to annotate endothelial cells. Analysis of gene marker expression to annotate endothelial cells

Supplementary Figure 18. Gene marker expression to annotate pericytes. Analysis of gene marker expression to annotate pericytes

**Supplementary Figure 19. Expansion of transdifferentiated heterophil cells in the spleens of *RAG1*-deficient chickens.** The proportion and absolute number of CD45⁺Bu-1⁻KUL01⁺ transdifferentiated heterophil cells were compared between the spleens of 1-week-old wild-type and *RAG1*-deficient chickens. Both the proportion and absolute number of heterophil cells were significantly increased in *RAG1*-deficient chickens compared with wild-type controls.

**Supplementary Figure 20. Unsupervised re-clustering of candidate heterophil and macrophage clusters.** Candidate heterophil clusters were re-clustered together with classical macrophage clusters across progressively increasing clustering resolutions (increasing numbers of subclusters) to assess their transcriptional relationships.

**Supplementary Figure 21. Metabolic and translational gene signatures of heterophil subsets.** Heatmaps displaying the expression of genes associated with (A) translation and (B) oxidative phosphorylation (OXPHOS), which are significantly upregulated in the H4, and H5 subsets. (C) Expression of cholesterol and fatty acid synthesis genes, uniquely enriched in the H4 and H5 populations

Supplementary Figure 22. Gene signatures of transdifferentiated heterophil subsets. Expression profiles of (A) M2 macrophage-associated genes enriched in H2, (B) vascular-interaction genes enriched in H3, and (C) T-cell-associated genes indicating cross-lineage transdifferentiation in H7.

**Supplementary Figure 23. Expression of genes associated with immature heterophil subsets.** Feature plots illustrating the expression level of the immature heterophil markers, (A) *NFE2*, (B) *PBX1*, and (C) *TAL1*.

Supplementary Table 1. List of oligonucleotide sequences used in this study

| ID | Sequence (5’→3’) | Usage |
| --- | --- | --- |
| *RAG1* seq F | AAGCAACTTTGCCGCATCTG | Genotyping |
| *RAG1* seq R | ACACGTTGTTTCCACTGGGT | Genotyping |
| *RAG1* donor F | GGAAGAAAGCGAAAGGAGCG | Genotyping |
| qRT *CSF3R* F | AGCAACCGAAGTCATCCTACTG | qRT PCR |
| qRT *CSF3R* R | CTGCCACTCCAGCACATAGG | qRT PCR |
| qRT *MMP9* F | CCCTATGTACAGCTACGTCCAG | qRT PCR |
| qRT *MMP9* R | CAGAGCCACGACCATAGAGG | qRT PCR |
| qRT *CYBB* F | TCGTCTCGGGCAAACATGG | qRT PCR |
| qRT *CYBB* R | TAACCCCAGCCACACAAGAAC | qRT PCR |
| qRT *NOS2* F | CCAAGCCGTGCATTCTTATTGG | qRT PCR |
| qRT *NOS2* R | TGTCACCACCTTTGATCCCTTTC | qRT PCR |

**Supplementary Table 2**. Antibodies panel used for staining of chicken heterophils

| **Primary antibody** | **Isotype control** | **Secondary antibody** |
| --- | --- | --- |
| Bu-1-FITC | Mouse IgG1-FITC | - |
| KUL01-AF647 | Mouse IgG1-AF647 | - |
| CD45-BIOT | Mouse IgM-BIOT | Streptavidin-Bv421 |
